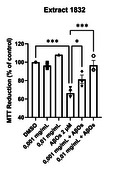# Exploring the potential neuroprotective properties of fungi living in extreme environments in a cell model of Alzheimer's disease: effects of Antarctic fungi extracts on cell viability and of Fomannoxin, a molecule produced by an Andean‐Patagonian fungus, on the viability and mitochondrial network of primary hippocampal neurons

**DOI:** 10.1002/alz70855_102844

**Published:** 2025-12-23

**Authors:** Matías Ignacio Lantadilla Brito, Jonhatan Gomez, Carol D SanMartín, Andrea Cristina Paula‐Lima, Jaime Cabrera‐Pardo

**Affiliations:** ^1^ Universidad de Chile, Santiago, Santiago, Chile; ^2^ Universidad del Bio‐Bio, Concepción, Concepción, Chile

## Abstract

**Background:**

Alzheimer's disease is related to hippocampal neurodegeneration induced by toxic soluble oligomers of beta‐amyloid peptides (AβOs) with molecular mechanisms such as increased intracellular calcium concentration, mitochondrial fragmentation, and mitochondrial ROS production. Fungus that inhabit extreme environments of low temperatures, high radiation levels, high dryness, and high‐speed winds, like Antarctica and Andean‐Patagonia, achieve their adaptation by producing unique secondary metabolites. Some of these molecules have bioactive effects and may exhibit potential neuroprotective effects.

**Method:**

Fungus extracts were provided by the Applied and Sustainable Chemistry Laboratory (LabQAS), Universidad del Bio‐Bio and R/S enantiomers of fommanoxin (FMX) were synthesized by Enamine (Kyiv, UKR). The effects of different Antarctic fungus extract (≈15) on the cell viability of primary rat hippocampal cultures (15 DIV) at different concentrations were tested. After 24 hours of incubation, the viability was measured using the MTT assay. Cultures were treated with fungus extracts at non‐lethal concentrations or fommanoxin for neuroprotection assays and co‐incubated 2 µM AβOs over 24 hours, followed by an MTT assay. For immunofluorescence, FMX 10 µM of both enantiomers were co‐incubated with 1 µM AβOs over 24 hours, followed by fixation and labeling with anti‐mHsp70. For calcium signals, 0,01 mg/mL of extract 1832 was preincubated over 24 hours, then loaded with Fluo4‐AM, and 500 nM AβOs were used for stimulation at the microscope.

**Result:**

Fommanoxin protected against the cytotoxic effects of AβOs without differences between the enantiomers, and preliminary results show a reduction in mitochondrial fragmentation. Fungus extracts don’t show toxicity at low concentrations, but some are toxic at high concentrations. Extracts 1832 and 1834 show significant protection against the cytotoxic effects of AβOs, and Extract 1832 exhibits a dose‐dependent neuroprotective effect. Preliminary extract 1832 also show a reduction in the increase of cytoplasmic concentrations of Ca^2+^.

**Conclusion:**

Fommanoxin, extracts 1832 and 1834 have neuroprotection against AβOs toxicity. In addition and preliminary, FMX reduces the mitochondrial network fragmentation, and extract 1832 reduces the increase of cytoplasmic concentrations of Ca^2+^, both effects induced by AβOs. The study of antarctical fungi extracts must continue to determine the possible molecular mechanisms involved in neuroprotection.